# From Industry to Dentistry: A Comprehensive Review of Zeolite as a Next-Generation Multifunctional Filler for Enhanced Mechanical Reinforcement and Antimicrobial Efficacy

**DOI:** 10.3390/dj13110540

**Published:** 2025-11-14

**Authors:** Sohaib Fadhil Mohammed, Mohd Firdaus Yhaya, Abdul Fattah Nongman, Matheel Al-Rawas, Marwan N. Arbilei, Tahir Yusuf Noorani

**Affiliations:** 1 Department of Operative Dentistry, College of Dentistry, University of Anbar, Baghdad 55431, Iraq; 2 Biomaterials Synthesis Laboratory, School of Dental Sciences, Universiti Sains Malaysia, Health Campus, Kubang Kerian 16150, Kelantan, Malaysia; afattahnongman@gmail.com; 3 Prosthodontic Unit, School of Dental Sciences, Universiti Sains Malaysia, Kota Bharu 16150, Kelantan, Malaysia; matheel@usm.my; 4 College of Biomedical Engineering, University of Technology, Baghdad 10066, Iraq; marwan.n.arbilei@uotechnology.edu.iq; 5 Conservative Dentistry Unit, School of Dental Sciences, Universiti Sains Malaysia, Health Campus, Kota Bharu 16150, Kelantan, Malaysia

**Keywords:** zeolites, dental materials, antimicrobial properties, remineralization, metal-modified zeolites

## Abstract

Zeolites are becoming potentially important multifunctional fillers in dentistry, providing a distinctive blend of mechanical reinforcement, remineralization, and antimicrobial properties. Their crystalline aluminosilicate frameworks offer ion-exchange capacity, the controlled release of therapeutic ions (Ag^+^, Zn^2+^, Ca^2+^, Sr^2+^, Cu^2+^), and compatibility with various dental composites. Sustainable and cost-effective zeolite production has become possible due to recent developments in synthetic strategies. These include the valorization of industrial and agricultural residues that are abundant in Si and Al. The incorporation of zeolites into dental adhesives, restorative composites, glass ionomer cements, root canal sealers, prosthetic materials, and implant coatings has been shown to improve mechanical stability and remineralization potential, and enhance antibacterial protection. The unique advantage of zeolites in integrating multifunctionality within a single system is emphasized when compared with other fillers, such as hydroxyapatite nanoparticles and bioactive glass. Nevertheless, obstacles persist with respect to clinical validation, regulatory pathways, and long-term biocompatibility. This review critically assesses the structure–function relationships, synthesis strategies, and dental applications of zeolites, while also delineating future perspectives for their translation into clinically approved, sustainable dental biomaterials.

## 1. Introduction

Dental caries is a widespread chronic disease that affects people of all age groups, especially those from low socio-economic backgrounds. At present, the research is mainly focused on developing improved dental materials that demonstrate better mechanical strength while fighting microorganisms, since composite restorations are persistently prone to secondary caries. Zeolites have gained attention as promising additives in dental composites. The crystalline structure of zeolites provides a hydrous compound consisting of aluminosilicates, which is organized into three-dimensional network structures with uniform dimensions for pores, channels, and cavities. In addition, synthetic zeolites are market leaders for advanced applications because their natural mineral composition allows for precise structural control and molecular purity [1]. These crystalline aluminosilicates form a three-dimensional porous framework (0.3–1.5 nm) with a high surface area and excellent ion-exchange capacity [2]. The extensive testing of various zeolites confirmed their favorable biological interaction properties. Human laboratory tests of Faujasite (FAU), Linde Type A (LTA), and ZSM-5 (MFI) zeolites indicated their minimal toxicity effects and reduced tissue inflammation, together with excellent tissue compatibility [3]. The FAU zeolite framework operates smoothly within oral tissues because of its proven performance in dental drug delivery systems. The clinical value of LTA zeolites remains powerful because of their two main beneficial characteristics; they serve as drug delivery systems and implant surface antimicrobial layers. Incorporating zeolites into dental composites can therefore reduce biofilm formation, enhance mineralization, and strengthen the composite–tooth interface. The restoration–tooth contact area triggers secondary caries, which results in restoration failure because this specific location needs antibacterial and remineralizing agents [4]. The antimicrobial action of zeolites stems from medicinal ions combined with physical properties that strengthen the aluminosilicate crystal structure. Zeolites have a specific system that results in the gradual release of essential mineral ions, including calcium (Ca^2+^), zinc (Zn^2+^), silver (Ag^2+^), and copper (Cu^2+^). Oral mineral saturation occurs through the combination of restorative ions with antimicrobial qualities. The hydroxyapatite structures inside dental materials respond to both Calcium Aluminosilicate Zeolite (CAU) and LTA zeolite systems by using retained calcium ions [5]. Thus, the mineralization process allows for both frequent composite repairs and resistance against dental caries. Furthermore, zeolites prevent biofilm development. *Gram-negative bacteria* growth is blocked by Zeolite X coatings, while these coatings allow for Gram-positive bacteria to form strains that dental professionals fail to identify in oral settings. Zeolite X releases calcium and sodium ions, which act as antibacterial agents, preventing bacterial attachment and subsequent caries formation. By combining hydroxyapatite with zeolites, their compatibility with dental tissue and their therapeutic benefits are enhanced [6].

Recent advances in surface functionalization, such as silane coupling and bioactive molecule incorporation, have further improved their integration into resin matrices and enhanced biological performance. This review introduces the structural and chemical features of zeolites relevant to dentistry, followed by a discussion of their antibacterial and remineralization mechanisms. It then explores strategies for surface modification and composite integration, reviews biological and mechanical outcomes, and highlights current challenges. Finally, future perspectives are outlined for advancing zeolite-based dental materials towards clinical application.

### 1.1. Origin, Formation, and Structure of Zeolites

**Figure 1:**
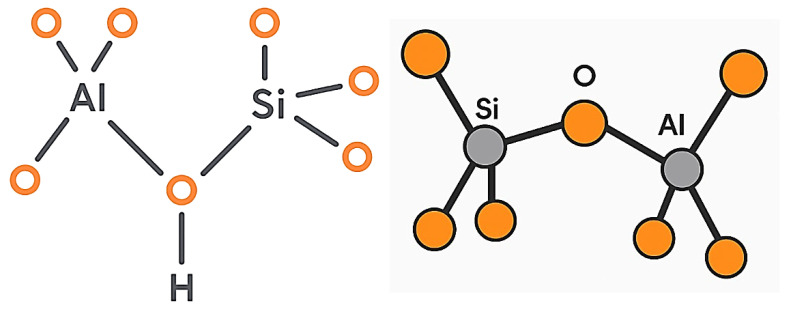
Tetrahedral arrangement of Al-O and Si-O in a zeolite crystal structure formed through volcanic processes [16].

Zeolites represent crystalline aluminosilicate materials characterized by unique microporous arrangements that scientists have studied due to their exceptional physicochemical properties in geological research, along with their industrial applications and biomedical use. The origin of zeolite formation depends on both natural geological phenomena and artificial chemical methods. Violent volcanic events create most naturally occurring zeolites because tectonic plate collisions cause magma eruption that breaks apart crustal segments and generates enormous flows of superheated fuels [7]. Under the influence of water, along with dissolved marine minerals, volcanic lava transforms its original disorganized volcanic content into well-structured crystalline zeolite structures (Figure 1) over the course of an extended geochemical process [8]. The identification “zeolite” comes from the Greek words “zeo” (to boil) and “lithos” (stone), and mineralogist Axel Fredrik Cronstedt first introduced this term in 1756. Through thermal heating exams, he detected mineral steam emissions, which demonstrated their water retention capacity during thermal initiation [9]. The structures of zeolites display microporous frameworks with ordered arrangements making up pores measuring from 3 to 10 Ångströms [10]. The framework design at the nanoscale dimensions grants zeolites the ability to perform both potent ion swapping and precise molecular uptake, which makes them suitable for carrying and swapping antimicrobial metal ions like silver (Ag^+^) and zinc (Zn^2+^) [11]. The zeolite framework operates as a permanent three-dimensional network system through the oxygen atom bonds between tetrahedrally arranged silicon and aluminum molecules. The framework structure incorporates both phosphorus atoms and aluminum and silicon atoms [12]. The tetrahedral configuration of aluminum and silicon framework atoms develops a negative charge that neutralizes through mobile cations Na^+^, K^+^, and Ca^2+^, which spread across the framework channels. Zeolites retain their structural stability after heating and drying because their hydration recovery process does not harm the framework. Zeolites form a material grouping that includes both natural and synthetic products. Natural zeolites such as clinoptilolite, mordenite, and chabazite acquire their chemical properties at geological formation sites through long geological development processes. Research laboratories use controlled engineering to design zeolites for specialized industrial and biomedical applications through precise chemical profile and porous attribute and functional response management [13]. In addition, the medical and dental sector have achieved major strides in contemporary material science because of their utilization of zeolite. Zeolites are utilized in dental composites and cements, and as regenerative biomaterials because of their targeted ion delivery, good tissue integration, and extended antimicrobial effects. Zeolites enhance oral healthcare treatment through improved dental structure adhesion and resistance against microorganisms [14]. The various framework types found in zeolites create difficulties in their correct classification. Zeolites surpass traditional crystalline solid definitions because of their extensive varied framework designs according to Baerlocher and colleagues [15]. The International Mineralogical Association’s Commission on New Minerals and Mineral Names implemented a new classification system in 1997 that defined framework matching as the standard for categorizing all zeolitic materials, while disregarding their silicon–aluminum content diversity [16,17]. The scientific community determined that zeolites match the crystalline material criteria since they use oxygen atoms bound to Si or Al atoms to build their tetrahedral framework and then create connected channels with enclosed areas. The continuous framework structure of zeolites develops structural breakdowns and terminal oxygen atoms at specific locations through the presence of OH^−^ or F^−^ groups. The extensive pore structure of zeolites enables three major functional properties—as molecular sieves, catalysts, and ion exchangers and antimicrobial agents—thereby supporting their broad application in science and technology [16].

### 1.2. Search Strategies and Data Sources

To guarantee the exhaustive coverage of studies pertinent to zeolites in dental biomaterials, the literature for this review was meticulously compiled from a variety of scientific databases. In addition to conducting searches on prominent publisher platforms such as MDPI, Elsevier, ACS Publications, Springer, and Wiley Online Library, PubMed, Scopus, Web of Science, and Google Scholar were the primary databases that were referenced.

Zeolites, dental biomaterials, antimicrobial zeolites, remineralization, ion-exchanged zeolites, silver zeolite, zinc zeolite, calcium zeolite, strontium zeolite, and copper zeolite were among the keywords and keyword combinations that were employed in the search. Articles that were pertinent and published between the years 1982 and 2025 were assessed. To offer a comprehensive and current review on the subject, both original research papers and review articles were included.

### 1.3. Utilization of Industrial and Agricultural Wastes Rich in Si and Al for Zeolite Synthesis

**Figure 2:**
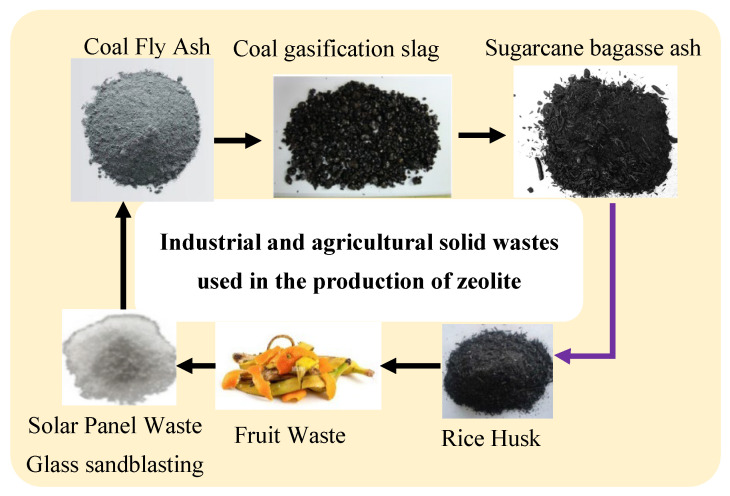
Industrial and agricultural solid wastes used in the production of zeolite [16].

Zeolites function as crystalline aluminosilicates that form microporous structures made of SiO_4_ and AlO_4_ tetrahedral networks, which link together with each other in a three-dimensional structural pattern. Industrial separation and ion exchange procedures heavily rely on zeolites because of their high cation-exchange capacity, together with uniform porosity and thermal resistance and chemical stability properties. Natural occurrences of zeolites present problems in industrial applications because their irregular compositions and lower purity levels, along with their framework imperfections, limit their operational performance. Scientists use synthetic zeolites to gain better control over zeolite production while defining both purity levels, framework construction methods, and pore sizes. Manufacturing synthetic zeolites through a production method necessitates the implementation of expensive processing substances, such as pure sodium silicate and aluminum hydroxide. Researchers have focused on developing economical zeolite production through secondary methods that utilize industrial waste containing large amounts of silica (SiO_2_) and alumina (Al_2_O_3_) (Figure 2). A detailed assessment of wastewater-based zeolite production methods appears in the following sections, where waste parameters meet the evaluation criteria for transformation and environmental impact analyses [18].

#### 1.3.1. Coal Fly Ash (CFA)

**Figure 3:**
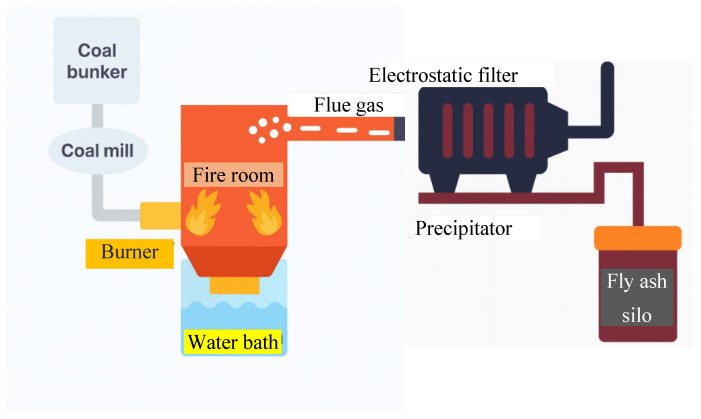
Schematic diagram of fly ash production in a coal-fired power plant. Pulverized coal is combusted in the fire room, producing flue gas that passes through an electrostatic filter and precipitator system. Fly ash is collected in a silo, while bottom ash settles into the furnace base [20].

Coal Fly Ash (CFA) is a fine particulate waste generated during the combustion of pulverized coal in thermal power plants. Due to the extensive global reliance on coal-fired power for electricity production, CFA has become one of the most abundant industrial solid wastes worldwide. During the combustion process, large volumes of flue gases are produced, which contain suspended ash particles. These particles are primarily captured by electrostatic precipitators or bag filters before being released into the atmosphere [19]. As depicted in Figure 3, coal is first ground in a coal mill and fed into the furnace via a burner. Inside the combustion chamber, or “fire room,” the coal is burnt at elevated temperatures, releasing thermal energy and producing ash.

The lighter portion of the ash becomes entrained in the flue gas stream, while the heavier bottom ash settles at the bottom of the chamber. The flue gas, carrying fly ash, is then passed through an electrostatic filter and precipitator, which efficiently captures the ash particles before venting the cleaned gas. CFA is mainly composed of amorphous aluminosilicate glass, along with crystalline phases including mullite (Al_2_SiO_2_), quartz (SiO_2_), hematite (Fe_2_O_3_), and magnetite (Fe_3_O_4_). Its particle size typically ranges from 1 to 100 μm, with high porosity (30–65%) and a specific surface area exceeding 10 m^2^/g [21].

The dominant chemical constituents are silicon dioxide (SiO_2_) and aluminum oxide (Al_2_O_3_), accompanied by smaller amounts of CaO, Fe_2_O_3_, MgO, and several trace heavy metals. Improper disposal of CFA can result in the leaching of toxic elements, contaminating nearby soil and water resources. To address both environmental and material value concerns, researchers have developed methods for converting CFA into synthetic zeolites through hydrothermal treatment or alkali fusion. These processes activate the aluminosilicate species, facilitating their reorganization into crystalline zeolite frameworks. Zeolite types such as Na-A, Na-X, and sodalite have been successfully synthesized from CFA, demonstrating high efficiency in ion exchange, especially for removing heavy metals and ammonium ions from wastewater systems [22].

#### 1.3.2. Coal Gasification Slag (CGS)

**Figure 4:**
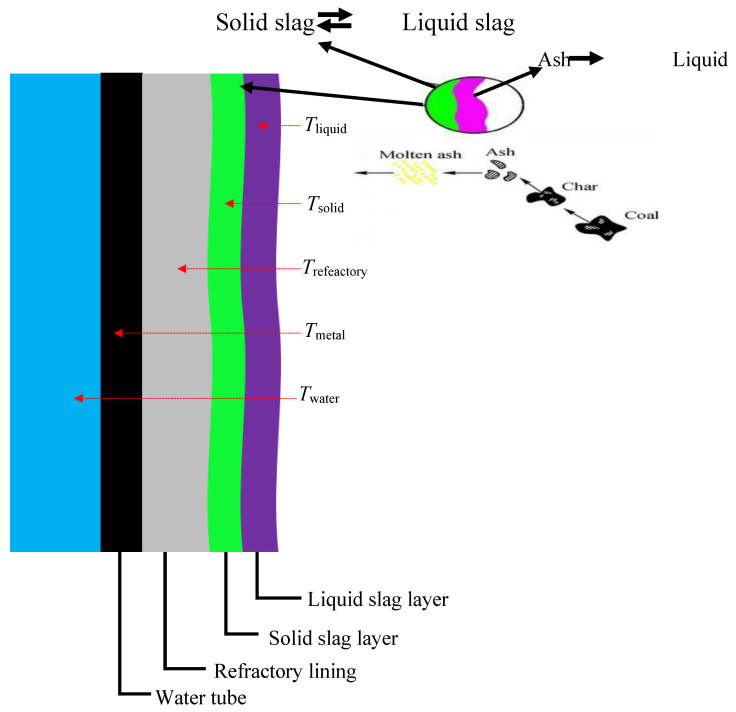
Slag formation during coal gasification. As coal pyrolyzes, mineral ash melts into molten slag, forming solid and liquid layers along the reactor wall. Thermal gradients (T_water to T_liquid) develop across the system [24].

Coal gasification is a thermochemical process used to convert solid coal into synthetic gas (syngas), which primarily consists of hydrogen (H_2_) and carbon monoxide (CO). This process takes place at extremely high temperatures in a reducing atmosphere, and during this conversion, a significant amount of non-combustible mineral matter in the coal melts to form slag. The molten material cools and solidifies into two main types: coarse slag (CS), which collects at the bottom of the gasifier, and fine slag (FS), which is carried by the gas stream and filtered out downstream [23]. The gasifier accepts coal and thermal decomposition and produces gases while producing char residue (Figure 4).

Rising temperatures within the coal space cause silica (SiO_2_) and alumina (Al_2_O_3_) mineral substances to melt into a thick liquid substance known as slag. Mining operators need to monitor two distinct reservoirs that develop inside the gasifier, since the inner liquid slag layer joins an outer solidified slag layer, to protect reactor linings while sustaining operational readiness. The chemical makeup of coal gasification slag contains 40–60% SiO_2_ and 20–30% Al_2_O_3_, with additional contents of CaO, Fe_2_O_3_, and MgO, together with trace elements Zn, Pb, Cu, Ni, Cr, and Mn. The specific composition of coal and gasifier operating factors determines the element concentrations between 37 µg/g and 4063 µg/g [25,26]. Virgin CGS shows high inertness because of its glassy matrix structure, but external activation processes such as alkaline fusion and mechanical milling can transform it. By combining slag with sodium hydroxide (NaOH) at high temperatures between 500 and 600 °C, the stable mineral structure decomposes into aluminates and soluble silicates. By applying hydrothermal treatment to these materials, three primary zeolite crystalline forms are obtained, including Na-P, together with sodalite and Na-X. The zeolites that are derived from CGS demonstrate advantageous features such as strong ion exchange properties with a high surface area, alongside good thermal stability. The zeolites obtained through this process demonstrate high effectiveness toward heavy metal elimination and ammonium absorption, along with gas adsorption properties, which make them useful for environmental and industrial needs [27].

#### 1.3.3. Rice Husk Ash (RHA)

Large quantities of rice husk develop as processing waste during the steps in rice milling agriculture. Rice husks contain 28% silica content, which allows for their use in many high-value applications in addition to their classification as a waste material. The production method for rice husk ash (RHA) requires controlled combustion heating ranging from 550 to 800 °C. Controlling combustion processes enables the transformation of ash materials into a product that maintains 85–98% amorphous silica content [28]. RHA demonstrates special properties which establish it as an optimal starting material for zeolite production. RHA contains three main characteristics: it is lightweight, with an extensive surface area and pore structure network. RHA converts into zeolites of Na-A, Na-X and ZSM-5 types when activated using combined sodium hydroxide alkali and hydrothermal crystallization methods. Research studies have identified that the zeolitic materials produced through this synthesis method have three major characteristics, combining favorable ion exchange capabilities with high thermal resistance and effective absorption [29]. The use of RHA material results in both environmentally friendly effects and material-based advantages. The transformation approach reduces atmospheric emissions of air pollutants combined with greenhouse gases that typically emerge from agricultural residue fires in open fields. Rice husk conversion yields RHA through an environmental method of agricultural waste management. Transforming agricultural byproducts into RHA helps in reducing the volume of landfill waste, helping waste management systems to meet sustainability targets. RHA exists in various manufacturing applications because of its broad industrial usefulness. The use of phosphoric acid to convert RHA into zeolites enables the removal of heavy metals and organic contaminants during water treatment operations. Mutually beneficial pore structures and surface selectivity make these materials active catalysts which work well in petroleum refining and chemical synthesis reactions [30].

#### 1.3.4. Solar Panel Waste Glass and Sandblasting Waste

**Figure 5:**
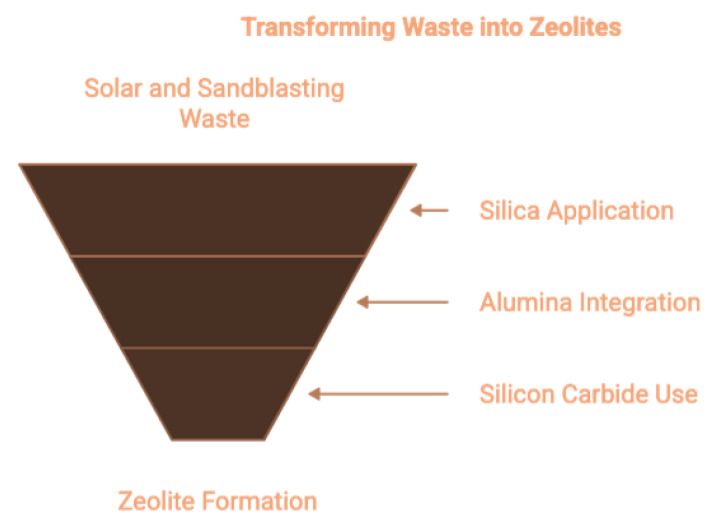
Conversion of solar and sandblasting waste into zeolites via silica, alumina, and silicon carbide recovery [16,19,21].

The continuous increase in solar power infrastructure installation has resulted in mounting solar panel waste accumulation rates. Photovoltaic panels comprise more than 80% of silica-based glass, with further components including alumina and silicon. The process of sandblasting waste (SW) creation after panel-cutting produces waste material that contains substantial amounts of silicon carbide together with unreacted silicon and alumina [31]. The high silicon and alumina contents in solar panel waste glass, along with SW, makes them suitable raw materials for producing zeolites (Figure 5). The conversion of these waste materials into zeolites Na-A, Na-P, and mesoporous variants is possible through alkaline fusion procedures and hydrothermal crystallization approaches. The waste cycle benefits from such reuse because it decreases e-waste impact while helping the circular economy [32].

#### 1.3.5. Sugarcane Bagasse Ash (SCBA)

**Figure 6:**
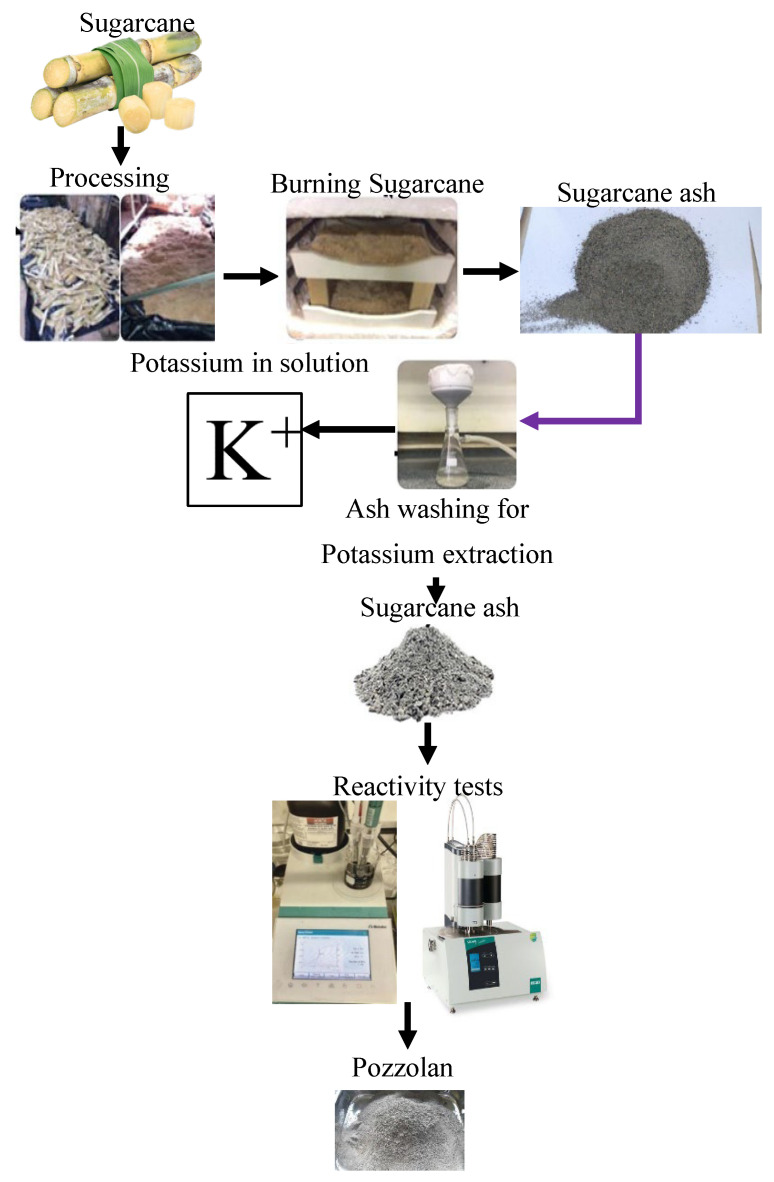
Process flow of sugarcane ash production and utilization, including potassium extraction and pozzolanic reactivity testing [36].

Bagasse is the fibrous residue that is present in sugar mills after the juice has been extracted from sugarcane (Figure 6). Bagasse is a byproduct of the sugarcane plant. The incineration of sugarcane bagasse leads to the formation of sugarcane bagasse ash (SCBA), which is considerably concentrated in silica (53–91%) and comprises 12–31% alumina, depending on the conditions under which it is burnt and the location of the burning [33]. Due to the fact that it has pozzolanic activity, SCBA is already being used in the cement and concrete construction industries. Nevertheless, recently, there has been increasing interest in its potential for the synthesis of zeolites. Through the process of alkali fusion, which is subsequently followed by hydrothermal treatment, it is feasible to effectively transform SCBA into zeolites that have a high surface area and the ability to exchange ions [34]. The materials that are formed as a result have the potential to be used in the treatment of industrial wastewater, the removal of heavy metals, and even gas adsorption systems. These applications need the materials to be manufactured [35].

#### 1.3.6. Fruit Waste Biomass

The processing of fruits leads to the production of large volumes of waste through the creation of peels, seeds, and skins, together with pulp residues. The remains from processed fruits, which mostly end up in landfills or serve as compost material, have been discovered to be promising raw materials to produce advanced functional components such as zeolites and activated carbon. Ash produced from organic sources attains its silica and carbon content after undergoing controlled thermal or chemical treatment, which are fundamental elements in the development of zeolites and porous adsorbents [37].

**Figure 7:**
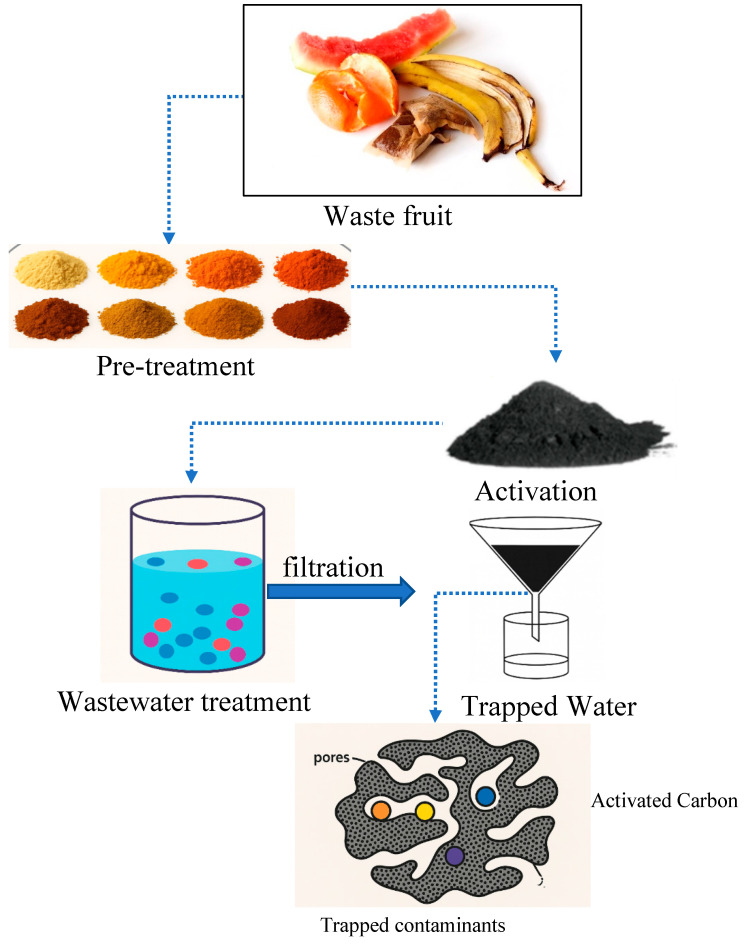
Fruit waste converted into activated carbon for contaminant removal in water treatment [38].

Figure 7 demonstrates that fruit waste follows a pre-treatment process which involves thermal activation via pyrolysis. During this process, the biomass becomes activated carbon through chemical conversion into a material with numerous small pores. The fabricated material shows exceptional pollutant absorption capabilities because it efficiently catches both organic substances and inorganic pollutants throughout its water purification operations [38]. The production of zeolite precursors through alkaline fusion reactions begins when ash from thermochemical fruit waste processing is converted into zeolites. Environmental researchers have developed this experimental method to transform biodegradable waste into value-added porous materials at a low cost for wastewater treatment applications, along with environmental remediation and catalytic purposes [39].

#### 1.3.7. Alternative Water Sources for Zeolite Synthesis

The production of synthetic zeolites through traditional methods needs deionized or distilled water as the starting material to achieve dependable material properties in the final product. Using large amounts of clean water in the synthesis increases both operational expense and environmental consequences, especially when working at a large scale. Various research efforts investigate alternative water sources, which consist of seawater and aluminum corrosion effluents and industrial brine and plasma electrolytic oxidation wastewater [16,40].

**Figure 8:**
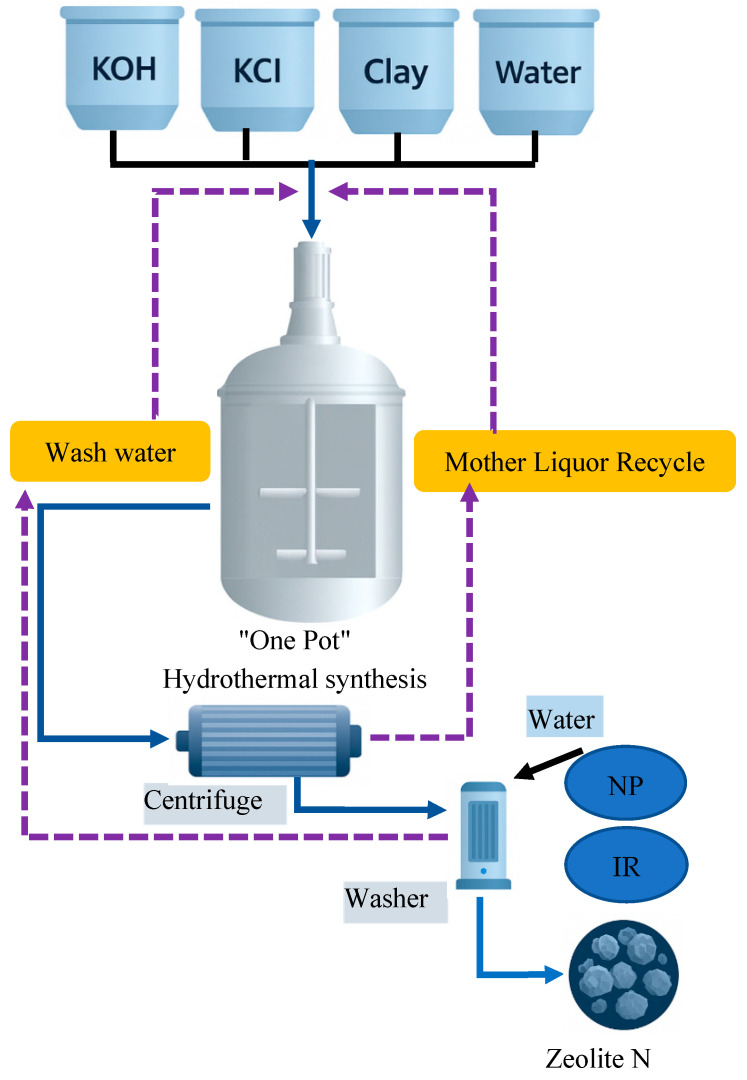
Schematic representation of a one-pot hydrothermal zeolite synthesis process utilizing recycled water streams. Wash water and mother liquor are recovered and reused to produce Zeolite N, enhancing both environmental and economic outcomes [41].

Scientists show that Na-A zeolite synthesis is possible through hydrothermal processing using coal fly ash and plasma electrolytic oxidation (PEO) wastewater in a single pot. The two key water recycling stages shown in Figure 8 allow the process to minimize its requirements for new water input during the mother liquor recycling and wash-water reuse stages. Na-A zeolites obtained through this method maintain a high cation exchange capacity alongside superior thermal and structural durability and low toxic element release properties for efficient environmental and industrial work [41,42]. This method decreases dependence on pure water while creating environmentally beneficial practices for recycling industrial waste liquids. The integrated method contributes to the development of zeolite production systems which promote sustainable resource efficiency alongside circular operations [43].

## 2. Synthetic Zeolites

Synthetic zeolites constitute an alternative which offers better control and adaptability compared to naturally occurring zeolites. Zeolites that form naturally require geological periods of time together with specific hydrothermal environments. Synthetic zeolites result from specific manufacturing approaches, which allow scientists total control over their structural and compositional properties as well as their operational characteristics. These materials exhibit valuable attributes for demanding application settings through their excellent purity and uniform morphology, alongside their changeable pore size and customizable chemical features [44]. Scientists can engineer synthetic zeolites with specific properties through the controlled manipulation of synthesis parameters, which include reaction temperature as well as pressure, while adjusting pH value alongside silica-to-alumina ratios. This versatility allows scientists to produce conventional zeolite structures alongside novel frameworks that provide unique capabilities. The following sections establish the fundamental synthetic methods through detailed explanations, including manufacturing processes, and their benefits, constraints, and industrial progress [45].

### 2.1. Hydrothermal Synthesis of Zeolites

**Figure 9:**
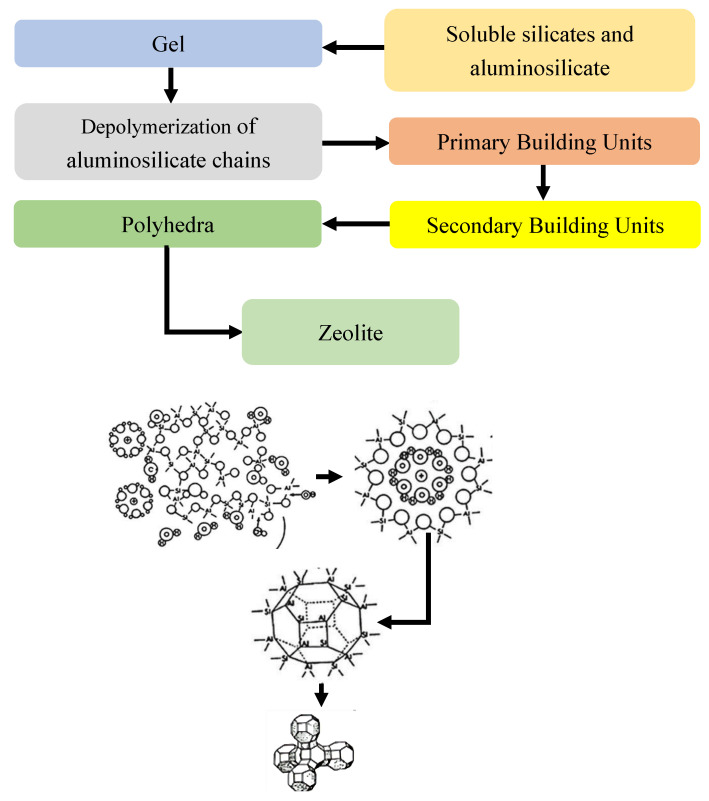
Diagram illustrating the hydrothermal synthesis pathway of zeolites [47].

Laboratory facilities utilize basic hydrothermal synthesis as the main method to create artificial zeolites. A reactive gel develops from combining silica and alumina in basic potassium hydroxide or sodium hydroxide solutions. The autoclave contains mixtures heated to ranges between 100 and 200 °C under water vapor pressure levels that control the pressure. The gelled material generates zeolite crystals because its environment supports the processes of both nucleation and crystal formation. The hydrothermal method succeeds in promoting precursor materials to form crystalline frameworks with perfect order through its established conditions. The silica and alumina precursor substances decompose into reactive constituents because of their alkalinity, which then transform into zeolite structures (Figure 9). Scientists use the dissolution–recrystallization method to build precise zeolite structures that have controlled precise pore frameworks. The alkaline solution allows precursor substances to restructure until both nucleation begins and crystal formation completes their development. The length of the crystallization period establishes how the end-product will appear, as longer reaction times produce bigger crystalline shapes with increased crystalline properties. Both faster reaction kinetics and thermodynamic selection of stable phases occur when the temperature rises, though these conditions might replace kinetically produced crystal structures. The control handed to researchers through hydrothermal synthesis enables the synthesis of zeolites for industrial applications by modifying reagent composition and pH as well as reaction temperature and duration [46,47].

Using hydrothermal synthesis allows zeolites to achieve highly crystalline structures with well-defined crystal arrangements. Multiple challenges continuously affect the method because it requires excessive energy consumption and produces long cycles and limited manufacturing capabilities. Reformulated hydrothermal approaches through various modifications were developed to remedy the existing shortcomings. The microwave-assisted hydrothermal method enables scientists to produce both speedier crystal development and uniform small crystals by improving reaction speeds [48,49]. The alkali fusion–hydrothermal method starts by fusing fly ash and clay materials to increase their reactivity and expand waste usage in zeolite manufacturing [50]. The synthesis process, using ultrasound and hydrothermal conditions together with microwave-digested alkali fusion, allows scientists better control of dissolution processes and nucleation dynamics to achieve efficient and quick synthesis [51,52].

### 2.2. Ionothermal Synthesis

**Figure 10:**
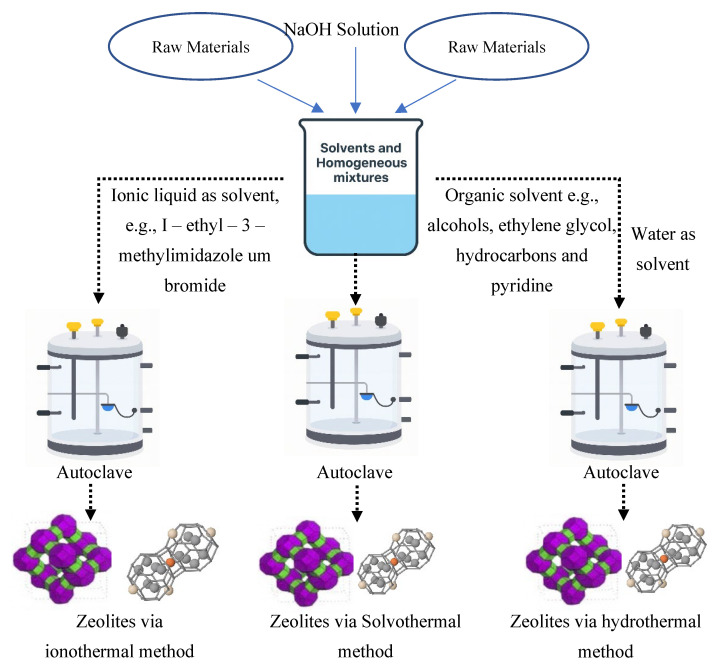
Schematic representation of the solvothermal synthesis process, including ionothermal and hydrothermal variants [55].

Ionothermal synthesis introduces a transformative approach in which ionic liquids or deep eutectic solvents replace traditional water-based solvents (Figure 10). These substances not only act as reaction media but also serve as structure-directing agents, reducing the need for additional templating chemicals. The low vapor pressure and high thermal stability of ionic liquids make them ideal for high-temperature syntheses that yield complex and highly ordered zeolite frameworks [53,54].

Ionothermal methods have successfully produced zeolites with unique topologies, particularly germanosilicates. However, the high cost and potential toxicity of ionic liquids remain challenging. Additionally, the slow crystallization rates observed in many ionothermal systems necessitate extended synthesis times, limiting large-scale application. Ongoing research focuses on identifying green, cost-effective ionic liquids to improve the method’s sustainability and practicality [55,56].

### 2.3. Solvothermal Method

**Figure 11:**
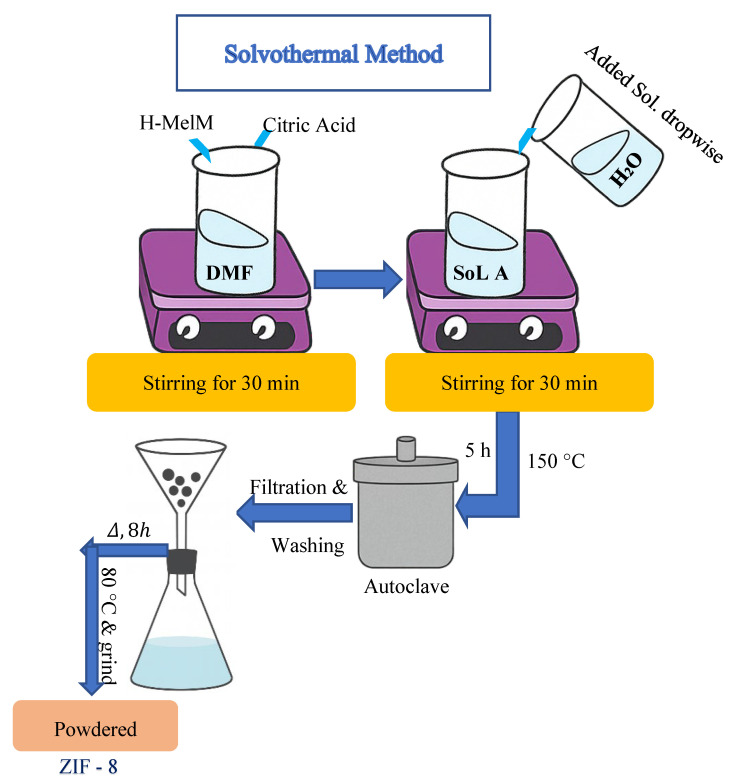
Solvothermal method [59].

Replacements for regular hydrothermal synthesis occur through the solvothermal method by switching traditional water-based solutions with organic solvents such as ethanol, methanol, and ethylene glycol. The use of organic solvents during solvothermal synthesis affects the behavior of solubility and diffusion and nucleation, which enables improved control over zeolite crystals and their morphology and porosity (Figure 11). The vapor pressure differences between organic solvents create autogenous pressure environments during solvothermal conditions that modify the crystallization conditions [57,58].

Through the solvothermal method, researchers achieve a high degree of control over the creation of hierarchical structures with hybrid zeolites. One drawback of this procedure exists in its lengthy reaction duration together with the detrimental environmental effects caused by employing organic solvents [59]. Waste treatment systems and solvent recycling initiatives need development for the process to qualify as environmentally sustainable. The solvothermal methodology continues as an essential method for fabricating advanced zeolite materials that perform specific catalytic functions and have adsorption-based applications [60,61].

### 2.4. Alkali Fusion and Leaching Method

**Figure 12:**
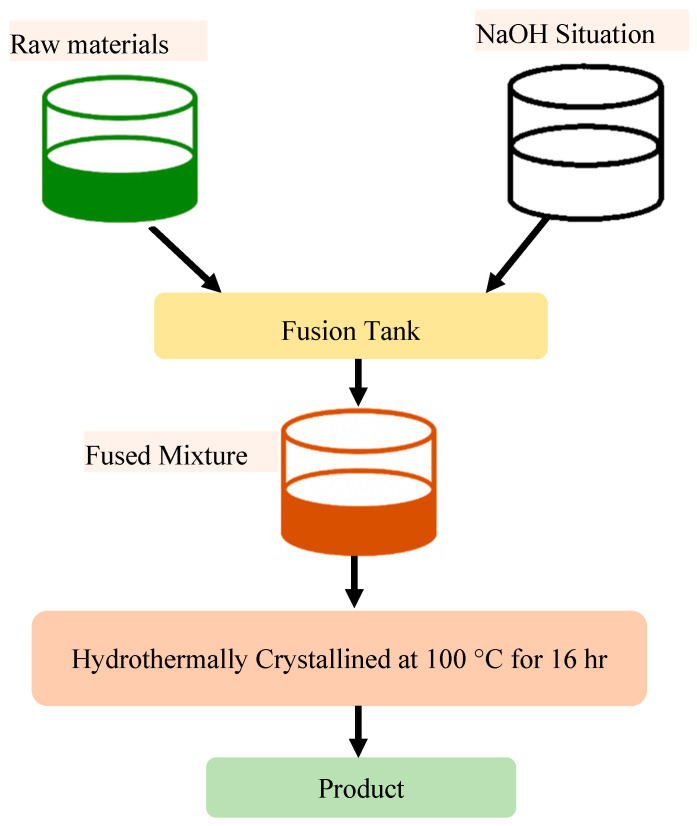
Zeolite synthesis via the alkali fusion route: fusion of raw materials with alkali followed by hydrothermal crystallization [55].

Inert or low-reactivity materials, such as fly ash and slag, may be converted into active zeolite precursors through the use of the alkali fusion process, which is especially well-suited for this purpose. To accomplish this procedure, the raw material is combined with sodium hydroxide and then heated to temperatures that are higher than 800 degrees Celsius (Figure 12). During this fusion stage, soluble sodium silicates and aluminates are produced. These aluminates and silicates are then cooled, ground, and subjected to further processing by hydrothermal crystallization to create zeolite structures [62,63].

Alkali leaching is an alternate method that involves the use of alkaline solutions for the purpose of extracting reactive components from solid waste materials. This method ensures that the essential ratio of silica to alumina is maintained. Following the extraction process, this solution is used as a precursor for the crystallization of zeolite. Factors influencing the efficiency of both methods include the composition of the starting material, the fusion or leaching temperature, and the concentration of the alkaline reagent [64]. The production of high-performance zeolites from industrial leftovers may be accomplished in a sustainable manner via the use of these technologies, which are particularly useful for recovering value from waste [65].

### 2.5. Sol–Gel Method

**Figure 13:**
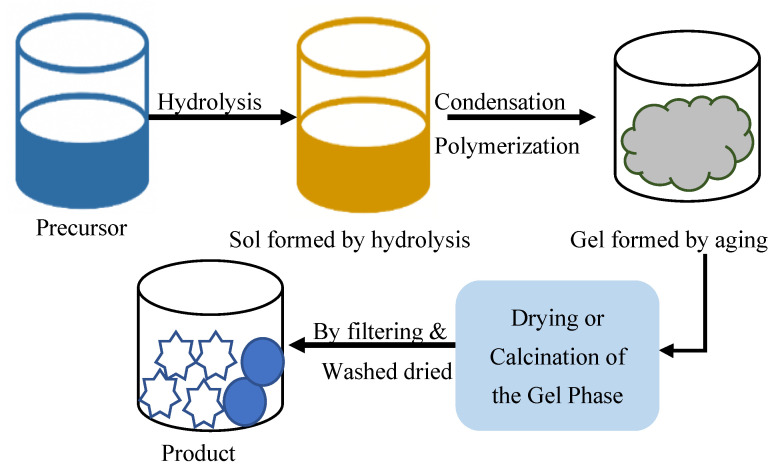
Flowchart of the sol–gel method for zeolite synthesis from colloidal suspensions [55].

The sol–gel approach offers a low-temperature method for the production of zeolites that have characteristics that are very consistent and can be adjusted to a certain degree. During this procedure, a sol, which is composed of colloidal silica and alumina particles in solution, is converted into a gel by means of hydrolysis and condensation processes (Figure 13). Calcination is then applied to this gel after it has been dried, which results in the formation of the crystalline zeolite structure [66].

The sol–gel method enables the effective monitoring of properties such as porosity alongside surface area and particle size distribution, so it is an effective way to fabricate zeolite films and nanostructures [67]. This substance receives limited industrial application because precursor expenses are high, and manufacturing demands strict process management. The high-value applications of zeolite made through this method extend to membrane creation and drug delivery alongside optical technologies [68].

### 2.6. Microwave-Assisted Synthesis

**Figure 14:**
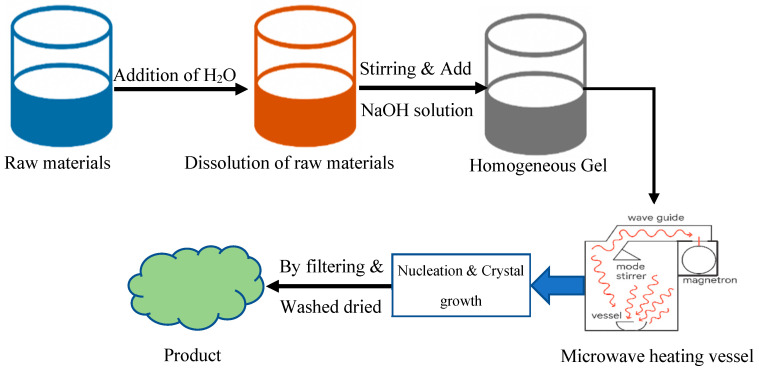
Microwave-assisted synthesis system showing rapid zeolite formation through dielectric heating [55].

The combination of microwave-assisted synthesis allows for the manufacturing of zeolites with better energy efficiency. The application of microwave radiation during this approach enables the continuous controlled heating of reaction solutions (Figure 14). The faster crystallization rate improves the phase purity standards of the system. The solution temperature rises quickly because its electromagnetic energy interacts with both molecules bearing dual polarities and ion-related substances found in the solution [69,70,71].

This process is especially useful when combined with other procedures, such as those using hydrothermal or alkali fusion, which are also beneficial in this context. The microwave-assisted synthesis method allows for quicker scale-up and better throughput, since it significantly cuts down on the amount of time needed for reactions and results in the production of smaller crystals with narrower size dispersion [72,73]. Despite this, maintaining temperature and pressure control in closed microwave systems continues to be a difficult task, especially when dealing with volatile solvents or components with a high boiling point [74].

### 2.7. Ultrasound Energy Method

The production of acoustic cavitation in a liquid reaction medium reaches its peak with high-frequency sound waves functioning within the 20 kHz to 2 MHz frequency range [75]. The growth and explosive disintegration of microscopic bubbles are the causes of cavitation, which is induced by ultrasound [76]. The secondary nucleation rates and crystal purity can also be enhanced by cavitation during the cooling crystallization process [77]. The ultrasound energy method provides a tunable zeolite synthesis due to the diverse traditional and emerging applications of synthetic zeolites. Zeolites synthesized via ultrasound-assisted methods exhibit superior physicochemical properties compared to their conventionally prepared counterparts: enhanced crystallinity, significantly reduced particle diameters with narrower size distributions, and remarkably uniform morphological characteristics. These materials demonstrate exceptional textural properties, including optimized surface areas, tailored pore architectures, and enhanced accessibility to active sites—attributes that directly translate to superior performance in catalytic, adsorption, and ion exchange applications [78]. Nevertheless, the ultrasound method is also combined with other conventional methods to enhance the efficiency of the development of zeolite materials. For example, the SSZ-13 zeolite was identified as an effective catalyst with long-term activity. However, the industry implementation was hampered by the lengthy crystallization period. Mu et al. (2017) reported a sonochemical-assisted method for pre-treatment prior to hydrothermal crystallization in the synthesis of SSZ-13 zeolite [79]. This method reduced the time required for zeolite synthesis. The alkaline treatment phase was suggested to enhance the potential of ultrasound irradiation of zeolite Y [80].

## 3. Zeolites for Advanced Dental Applications

### 3.1. Functionalized Zeolite Frameworks for Dental Biomaterials

The application of zeolite compounds in dentistry expands when elements and their derivatives attach precisely to the materials. Aluminosilicate frameworks used by engineers allow for biomaterials to be developed for dental practices through the precise insertion of selective element ions to create specialized material properties. Strontium zeolite and silver zeolite serve as primary dental treatment compounds together with zinc zeolite and calcium zeolite because their ion exchange and sustained release functions are achievable [81].

#### 3.1.1. Silver Zeolite (AgZ): Superior Antimicrobial Functionality

The antimicrobial properties of silver zeolite frameworks stem from the controlled release of silver species. Different antimicrobial mechanisms operate through silver ions (Ag^+^) and charged silver clusters (Ag_n_^m+^), as well as metallic silver nano ions, which exist in multiple forms in the zeolite framework. The placement and quantity of silver within the zeolite framework depends heavily on the framework typology and silicon-to-aluminum ratio because these factors define the available charges for cation swapping. The exchange properties of silver zeolite directly oppose its stability levels in solution. Zeolites possessing higher cation-exchange capacities will rapidly release silver ions from their framework for cation exchange or aggregation, therefore achieving enhanced antimicrobial properties yet inducing shorter protection periods. The quantity of silver ion release depends directly on the specific surface area of zeolite, while pH environment affects rates through acceleration in acidic conditions [82,83,84]. A silver-ion antimicrobial mechanism comprises four distinct biological routes, which include silver-ion environmental distribution, bacterial cell dehydration, bacterial membrane penetration and the inhibition of DNA replication and cellular respiration. Silver-containing aluminosilicate antimicrobial performance depends heavily on the zeolite structural space (between micropores and mesopores) and physical state (powder, pellets, films), together with the presence of silver species (ionic silver or metal nanoparticles). Silver zeolite has received extensive laboratory testing to prove its superior biosafety; therefore, it has found widespread use in food preservation and medical device disinfection while showing promise in new dental materials that require controlled antimicrobial action to succeed clinically [85].

#### 3.1.2. Zinc Zeolite (ZnZ): Multifunctional Therapeutic Agent

There are two fundamental variants of zinc zeolites, which include zinc-cationic zeolites that exhibit zinc ions at exchange sites and zinc-oxide zeolites that contain zinc oxide species integrated within the zeolite framework. The stability of these materials remains exceptional because zinc provides strong stabilizing support to the metal–zeolite network structure, which benefits dental applications through increased structural resilience and functional durability [86]. The therapeutic features of zinc zeolites comprise powerful antibacterial capabilities together with substantial anti-inflammatory actions and osteogenic stimulation, therefore making these materials useful for dental implant and periodontal treatments. The antibacterial activity of zinc zeolites functions by gradually releasing zinc ions, which deter bacterial metabolism and cellular membrane functions, alongside releasing reactive oxygen species, including hydrogen peroxide and superoxide ions and hydroxyl radicals, which create oxidative stress in bacteria. Bones gain therapeutic benefits from zinc because it enables control of the inflammation pathways as well as facilitating bone matrix enzyme functioning due to its essential role in bone mineralization. The broad therapeutic capabilities of zinc zeolites make these materials superior candidates for dental biomaterials that manage to fight multiple oral healthcare problems simultaneously [87,88].

#### 3.1.3. Calcium Zeolite (CaZ): Remineralization Potential

Preparing calcium zeolite frameworks enables researchers to develop systems with specific particle sizes and design molecular sieve capabilities that release calcium ions in biological solutions. The dynamic oral environment triggers calcium zeolite function to serve as an ion storage unit that releases calcium ions directly to the tooth surface, where remineralization processes begin. The calcium ions that are released find phosphate ions in saliva to recover and rebuild the hydroxyapatite crystals in dental structures, which reinforce areas compromised by demineralization caused by bacterial acid activity. Due to their ability to regenerate mineral ions, calcium zeolites serve as essential tools for the early-stage management of dental caries. The physical adsorption capabilities of the material are enhanced through the combination of calcium ions and zeolite frameworks because this improves the removal of harmful substances and bacterial byproducts from the oral environment [4,86]. Hydroxyapatite composites with zeolite function as alternative calcium-release platforms which show remineralization capabilities alongside enhanced characteristics of biocompatible integration with the natural tooth structure [89].

#### 3.1.4. Strontium Zeolite (SrZ): Enhanced Remineralization and Cellular Stimulation

Under physiological conditions, strontium zeolite functions as an advanced biomaterial that maintains prolonged Sr^2+^ ion release. Strontium ions that are released through physiological conditions exhibit a unique ability to take the place of calcium ions in dental hard tissue apatite crystal structures, thus creating modified hydroxyapatite structures that might have stronger acid-resistance properties. The cellular response to strontium shows direct benefits for human dental pulp stem cells because it induces both their multiplication and cell differentiation. Strontium zeolites serve as excellent material components in advanced dental products that simultaneously support remineralization and cell activation, making them useful for restorative and regenerative dental materials [86,88].

#### 3.1.5. Copper Zeolite (CuZ): Potent Antimicrobial and Adjunctive Agent

Due to their efficacious antimicrobial properties against both Gram-positive and Gram-negative oral pathogens, copper-modified zeolites (Cu^2+^-exchanged or CuO-supported) have garnered attention. The sustained release of Cu^2+^ ions is the source of their antibacterial activity. This release disrupts bacterial cell membranes, generates reactive oxygen species, and interferes with enzymatic pathways, resulting in microbial mortality. In contrast to silver zeolites, copper zeolites exhibit a substantial bactericidal effect while maintaining a lower cost and a broader accessibility. Furthermore, their synergistic combination with copper oxide (CuO) nanoparticles improves the antimicrobial spectrum and the durability of ion release. Potential endodontic materials, antimicrobial coatings, and restorative composites are also being investigated for dental applications. However, to prevent potential tissue irritation, it is necessary to optimize the concentration of copper ions to achieve an equilibrium between antibacterial efficacy and cytocompatibility [5,11]. Table 1 provides a summary of the ions, emphasizing the dental applications and main benefits of each one, in addition to zeolitic frameworks used with each ion.

### 3.2. Clinical Applications of Zeolites in Modern Dentistry

#### 3.2.1. Microbiological Applications: Addressing Antimicrobial Resistance

The growing antimicrobial resistance problem requires the prompt development of antibacterial alternatives independent of standard antibiotic functions. Antibacterial metal-functionalized zeolites consisting of Ag^+^, Zn^2+^, Cu^2+^, and Co^2+^ show promise as an antibacterial agent against oral resistant pathogens through simultaneous mechanisms that limit the development of antimicrobial resistance [11,92]. Inorganic material aluminosilicates demonstrate greater resistance than traditional antimicrobial agents because they resist chemical and thermal changes during the manufacturing processes of dental material blends [93]. The combination of silver-treated zeolites gained prominence due to its antibacterial properties, because silver shows broad antimicrobial effects, and zeolites’ microporosity and high surface area control silver particle release and achieve extended antibacterial protection. Laboratory evidence showed that faujasite (FAU) zeolite films containing stabilized silver nanoparticles destroyed Escherichia coli bacteria after 120 min of exposure, thus demonstrating swift antibacterial behavior [94,95]. A new generation of antibacterial methods includes NO-loaded zinc-exchange zeolites, which show two-fold bactericidal action by defending against Gram-negative and Gram-positive microbes. Scientists keep researching ideal silver concentrations, together with mechanisms that achieve peak antimicrobial activity while reducing unwanted silver deposit effects through zeolite systems which maximize the strength of therapy using minimum silver content [96].

#### 3.2.2. Dental Adhesive Systems: Enhanced Bonding and Antibacterial Properties

Zeolites serve as an essential ingredient in dental adhesive systems because they resolve major drawbacks that traditional formulations face regarding their restoration margin, secondary caries from bacterial penetration, and adhesive bond deterioration. The combination of zinc and silver into zeolites substantially enhances dental adhesive wettability, antibacterial action, and biocompatibility, which improves the long-term bond strength between dental substrates and resin materials. Zeolite imidazolate, a framework subclass of metal–organic materials with zeolite structures, delivers vital benefits to dental adhesives through better viscoelastic performance and stronger bond strength and greater thermal endurance [97,98,99,100]. The zinc ions embedded in these frameworks inhibit matrix metalloproteinase, which blocks dentine collagen fiber hydrolytic degradation, thus improving the resin–dentin interface’s structural integrity and resulting in a longer service life for bonded dental restorations. The micron-sized zeolite network added to adhesive systems creates three benefits by strongly connecting to dental surfaces and allowing for long-term drug delivery capabilities, which helps to maintain interface integrity in the oral environment [101].

#### 3.2.3. Restorative Materials: Multifunctional Zeolite-Enhanced Composites

The addition of zeolite to dental materials improves their antimicrobial resistance while simultaneously enhancing their physical characteristics. Resins containing zeolites show superior bacterial detachment performance compared to standard resin materials, thus preventing primary restoration failure. Notably, these materials demonstrate lower wettability levels, which signifies reduced water absorption and enhanced resistance against hydrolytic degradation that affects composite age [102]. Restorative materials that contain calcium zeolites continuously release calcium ions into the tooth structure, which act as a preventive mechanism against the development of caries in the area surrounding the restoration margins [103]. Lab studies confirmed that silver–zinc zeolite, when added to temporary filling materials, prevents Streptococcus species, particularly *S. pyogenes*, *S. pneumoniae*, *S. salivarius*, and *S. haematogenic*, from growing by carefully managing the silver–zinc ion release. Composite materials filled with zeolites exhibit improved mechanical properties due to their structural characteristics and particular combinations of zeolite types and surface treatments, which affect compressive strength, flexural strength, and wear resistance effectiveness during use in oral environments [104].

#### 3.2.4. Glass Ionomer Cements: Extended Antimicrobial Properties

Zeolites function as an antibacterial agent during the embedding of ion-embedded glass ionomer cements (GICs) by offering sustainable therapeutic antibacterial properties. The antibacterial inhibition of Streptococcus mutans bacteria increases in step with the addition of silver-incorporated zeolite (AgZ) to the samples. The antimicrobial protective properties of GICs become stronger when AgZ is present in the material composition. This effect occurs because silver ions are sustained during delivery beyond fluoride release, which occurs within 48 h. The inclusion of zinc-containing zeolites (ZnZ) in GICs leads to an effective pathogen-suppression capability against *E. coli*, *S. aureus*, *P. aeruginosa*, *B. subtilis*, and *Candida albicans*. The antimicrobial defensive capabilities of this material operate against various types of pathogens. The combination of antibacterial properties present in chlorhexidine-filled zeolite GIC formulations enables effective *S. mutans* targeting, which leads to better antimicrobial effects [88,105,106]. The combination of ZUT mixed with 0.2% AgZ and KT-308 GIC sealer demonstrates superior effectiveness in *Enterococcus faecalis* elimination when applied as an endodontic material. Research outcomes differ as an effect of experimental conditions coupled with the formulation types selected by researchers. When zeolite is added to GICs, it leads to the emergence of different mechanical properties. Different zeolite types, along with differing dosage percentages and end purposes of the solution, lead to different characteristics. ZUT demonstrates better strength binding than Ketac-Endo alone, without being negatively affected by other conditioning agents present in the sample [97,107]. A zeolite concentration of more than 3% results in reduced material mechanical properties, but the optimal strength occurs between 1% and 2% zeolite levels during compression testing. The laboratory results showed that adding one percent by weight of zeolite nanoparticles to chlorhexidine did not affect the mechanical properties of dental materials, which remained unchanged. The nanoparticles show the ability to maintain their antibacterial efficiency along with their mechanical features, according to the research findings [105,106].

#### 3.2.5. Mineral Trioxide Aggregate: Enhanced Antimicrobial Properties

Research shows that mineral trioxide aggregate accompanied by silver zeolite (AgZ) exhibits powerful antimicrobial effects to effectively combat clinically significant oral microorganisms. AgZ-modified MTA successfully inhibits major oral bacteria comprising the difficult-to-treat endodontic microbes *Enterococcus faecalis*, *Staphylococcus aureus*, and *Candida albicans*, yet does not prevent the growth of Prevotella intermedia and Actinomyces israelii bacteria [108]. The inhibitory activity of AgZ MTA increases proportionally to its concentration because an increase in silver ion production correlates with 0.2% AgZ MTA, showing weaker microbial effects than 2% AgZ MTA. The antimicrobial capabilities of MTA containing 2% AgZ were better than MTA with 2% chlorhexidine, thus demonstrating promising potential for the use of AgZ as an MTA additive [108,109]. The addition of zeolites to MTA affects several physical characteristics through alterations in setting time, water penetrability, and bond strength during injection, along with compressive strength. The setting time shortens with increasing amounts of AgZ in MTA, whereas water absorption reaches its lowest point when AgZ concentration reaches 2%. Ag-Zn zeolite composites weaken MTA tensile bond strength and compressive strength when added to the mix, potentially because zeolite porosity disrupts the normal hydration processes needed for proper MTA crystallization. The combination of antimicrobial property enhancements and physical modification effects requires zeolite concentration control in MTA solutions for certain clinical applications, according to research that identifies the optimal amounts to achieve therapeutic benefits and sufficient mechanical performance [110,111,112].

#### 3.2.6. Resin Cements: Selective Antimicrobial Efficacy

By incorporating zeolite into resin cements, the antibacterial properties of the cements against specific oral bacteria are powerfully enhanced. The antibacterial inhibition of Streptococcus mutans and Streptococcus mitis by separate quantities of silver zeolite (AgZ) and zinc zeolite (ZnZ) remains effective but limited activity was observed against Streptococcus salivarius and Streptococcus sanguis. The antibacterial effect of resin-based systems using silver–zinc zeolite does not follow the same pattern as glass ionomer cements, where higher concentrations produce better results. The materials demonstrate different ways in which they release substances and perform antimicrobial actions [104]. The incorporation of diazonium group-functionalized zeolites into resin-based composites leads to the preservation or improvement of their compressive and flexural strength properties. This specific modification method requires additional research to establish its complete impact on material performance stability and compatibility with biological tissue [103].

#### 3.2.7. Root Canal Irrigation Solutions: Alternative Antimicrobial Approach

Silver zeolite formulations at 2% AgZ concentration prove superior for antibacterial performance when used as root canal irrigation solutions in comparison to regular saline treatment. The antibacterial properties of these formulations prove less effective when compared to the standard endodontic irrigants, which include sodium hypochlorite 5%, chlorhexidine 2%, and octenidine 0.10%. The decreased efficiency of silver zeolite solutions may stem from the specific antibacterial resistance displayed by endodontic pathogens, including *Candida albicans*, *Staphylococcus aureus*, and *Enterococcus faecalis*, against silver-based antimicrobials. Research must continue to identify the full performance parameters of silver zeolite irrigation solutions, including their tissue-dissolving and smear-removal ability, along with detailed analyses of their compatibility with endodontic materials to define their versatility in endodontic practice [113].

#### 3.2.8. Acrylic Resin Prosthetic Materials: Sustained Antimicrobial Protection

Research proves that acrylic resins featuring silver zeolite show better antimicrobial effects against major oral bacteria *S. mutans*, *F. nucleatum*, and *C. albicans*. Acrylic resins in removable prosthetics rely on conventional chemically polymerized acrylic resin material, which absorbs water and encounters difficulties in reaching full polymerization, thus creating an optimal environment for microbial growth and biofilm development. The incorporation of silver zeolite in acrylic resins solves this concern through its ability to minimize *S. mutans*, *F. nucleatum*, and *C. albicans* attachment to PMMA surfaces for 45 to 60 days [114,115]. The addition of 2.5% silver–zinc zeolite (Ag-Zn-Ze) to PMMA shows adequate success in blocking *Candida albicans* and Streptococcus mutans growth, indicating that both AgZ and Ag-Zn-Ze are practical approaches for strengthening acrylic-based dental prosthetics’ antimicrobial properties. The amount of zeolite additives that are added becomes a crucial factor because excess content above 2.5% by weight leads to decreased impact strength and flexural strength in these materials. The particular thermosetting of acrylic resins QC20 and Lucitone 550 retain adequate flexural strength exceeding 65 MPa (as per ISO standards) after zeolite incorporation, thus demonstrating their suitability for antimicrobial denture use. The recommended amount of zeolite for balancing structural integrity with antimicrobial effects should stay below 4% weight, while 2% performs best for achieving both mechanical properties and antimicrobial efficacy. Oral health professionals view the addition of silver–zinc zeolites to prosthetic materials as an effective way to stop denture stomatitis and other oral infections while advancing prosthetic dentistry outcomes for patients [115,116,117,118].

#### 3.2.9. Non-Acrylic Resins and Ceramics: Enhanced Properties and Performance

Zeolites are used within numerous dental products, including both soft denture liners and high-performance all-ceramic prosthesis. The product line also contains acrylic polymers alongside other compounds. The incorporation of silver zeolite into soft liner formulations maintains the necessary viscoelastic characteristics for comfortable wear and enhances bacterial resistance against *Candida albicans* and *Gram-negative bacteria* [119]. The efficient integration of sodalite zeolites within ceramic materials makes them ideal for use in ceramic prosthetics. Their selective properties, along with their catalytic properties, make sodalite zeolites stand apart as an exceptional zeolite subtype. The flexural strength of specimens containing sodalite zeolites surpasses ISO regulations and makes these specimens ready for use in medical settings. Scientists found that ceramic materials achieve superior flexural strength and strength measures when zeolite infiltration precedes high-temperature sintering at 1600 degrees Celsius when compared to standard glass-infiltrated control samples. Sodalite zeolite molecules that infiltrate zirconia-toughened alumina (ZTA) act as superior ceramic materials compared to glass-infiltrated counterparts due to their higher elastic modulus and fracture toughness combinations. This is a noteworthy finding. Sodalite zeolite-infiltrated ceramics demonstrate unique mechanical properties with increased bond strength, flexural strength, Vickers hardness, fracture toughness, and Young’s modulus, which establishes them as favorable materials to succeed glass-infiltrated ZTA in high-performance dental prostheses under demanding occlusal forces [120,121,122,123].

#### 3.2.10. Dental Implants: Antimicrobial Surface Modifications

The main purpose of adopting zeolite applications in implant dentistry is the addition of antimicrobial coatings onto surfaces to block the development of peri-implantitis and enhance osseointegration. Research on this precise implementation remains scarce but demonstrates how titanium implants with silver zeolite coatings effectively eliminate methicillin-resistant Staphylococcus aureus (MRSA), which is a prevalent implant-related pathogen. The strong anti-infective properties of zeolite coatings, combined with their good compatibility with human body tissue, indicate significant potential applications in implant dentistry, particularly for subjects who face a high risk of peri-implant infections because of systemic health issues or poor oral care [124].

#### 3.2.11. Oral Medicine: Diagnostic Applications

The diagnostic functions of zeolites were established in oral medicine through their contribution to oral cancer detection. Oral squamous cell carcinoma remains the most widespread form of oral cancer, and is the sixth leading cancer worldwide, leading to the generation of volatile organic compounds that function as molecular cancer markers. Researchers used gas chromatography–mass spectrometry together with thin-film microextraction analysis equipped with Zeolite Socony Mobil-5 (ZSM-5)/Polydimethylsiloxane (PDMS) hybrid films to evaluate volatile organic compounds in oral squamous cell carcinoma patients, according to Shigeyama and colleagues [125]. Socony Mobil-5 zeolites served as a detection system which enabled the identification of tumor-specific biomarkers through a sampling technique, showing superior benefits to blood testing through economic screening, patient-friendly designs, and screening opportunities. Dentistry now utilizes zeolite materials for diagnostic and therapeutic functions through advanced applications beyond medicinal functions, leading experts to believe zeolite frameworks will lead to the future development of diagnostic–therapeutic systems [126,127,128].

### 3.3. Limitations, Controversies, Comparison with Other Fillers, and Future Research Directions

Zeolites have dental applications with compelling multifunctionality by means of ion exchange, sustained antimicrobial release, and remineralization. Nevertheless, it is imperative to address several concerns. At elevated concentrations, particularly when utilizing Ag- or Cu-exchange zeolites, dose-dependent cytotoxicity has been observed, with the toxicity being influenced by the target cell type, ion dose, release kinetics, and particle size [1].

Sustained ion release is advantageous due to its antimicrobial and remineralizing properties; however, unregulated long-term release may result in systemic accumulation, tissue irritation, or oral microbiome disruption [129]. Zeolite incorporation can result in mechanical trade-offs in terms of materials performance, as it can reduce flexural strength and modify optical properties due to poor dispersion or excessive loading [130].

Another concern is the stability of zeolite frameworks under oral conditions, as they may be subject to ion exchange, partial degradation, or contamination in complex environments, such as biofilms or acidic challenges. Although Ca–Ag-zeolite formulations demonstrate encouraging physicochemical stability in vitro, their durability under cyclic pH fluctuations and repeated loading in vivo remains unconfirmed [81]. Furthermore, nanoscale zeolite granules present handling and safety concerns, necessitating more stringent occupational and manufacturing controls due to the potential for aggregation and inhalation exposure during the preparation process [3].

Beyond technical factors, regulatory uncertainty persists: metal-loaded zeolites remain in a gray area between medical devices and drug–device combinations, complicating approval pathways and delaying clinical translation [131]. Additionally, potential drawbacks such as color stability, long-term degradation, and biocompatibility concerns should be considered, as these may further limit the clinical acceptance of zeolite-based dental materials [81,130,131].

Several controversies also remain unresolved. The long-term ecological balance between antimicrobial efficacy and microbiome stability is not clear, and there is concern that extended ion release may encourage resistance or alter commensal microbial populations [4]. Additionally, heterogeneous in vitro models with varying cell types, bacterial strains, and media limit reproducibility and meaningful comparison across studies, underlining the need for standardized testing systems [3]. The development of waste-derived zeolites offers sustainability advantages but raises safety concerns regarding contaminants and heavy metals, requiring rigorous purification protocols [132].

Zeolites hold a unique position in comparison to other emerging fillers. A recent 2025 systematic review confirmed the clinical efficacy of bioactive glass (BAG) in reducing dentin hypersensitivity for up to 12 weeks, as it is well established for remineralization through hydroxycarbonate–apatite formation and Ca/Si/P release [133]. In contrast to Ag- or Cu-exchange zeolites, BAG’s antimicrobial effect is significantly weakened due to pH elevation. Additionally, its brittleness at high loadings can compromise mechanical properties, despite advancements in particle engineering [134]. Hydroxyapatite nanoparticles (nHA) are biomimetic and highly biocompatible, thereby facilitating osseointegration and remineralization. Their efficacy in implant coatings with enhanced bone response was emphasized in a 2025 review [135], and their protective function against enamel erosion was confirmed in another [136].

However, hydroxyapatite nanoparticles are incapable of exerting robust intrinsic antimicrobial activity unless they are altered, and they are confronted with the challenges of aggregation and dispersion. In contrast, zeolites are the only materials that combine multifunctional ion exchange, enabling the simultaneous application of antimicrobial (Ag, Zn), remineralizing (Ca, Sr), and therapeutic effects in a single system. Such functionality is achieved through the use of more controlled release profiles, such as the release of silver ions over a period of 30 days in GICs, compared to bioactive glass [129]. However, zeolites are subject to more intricate regulatory obstacles and lack long-term clinical validation, in contrast to bioactive glass and hydroxyapatite nanoparticles [2,133,134,135,136]. Table 2 provides a summary of the comparison, emphasizing the relative strengths and weaknesses of hydroxyapatite nanoparticles, bioactive glass, and zeolites.

To facilitate cross-study comparison and harmonize models, it is necessary to implement standardized, clinically pertinent testing protocols [3]. Safe therapeutic ion windows should be established, and systemic distribution should be assessed through comprehensive dose–response and protracted biocompatibility studies [1,129]. Scalable, cost-effective methods with stringent contaminant control should be the primary focus of synthesis advancements, particularly when employing waste feedstocks [132]. The classification and design of trials that meet the approval requirements will be clarified through early regulatory engagement [131]. The most critical aspect is the necessity of long-term clinical studies (≥2–5 years) to assess real-world outcomes, including caries prevention, restoration survival, peri-implant health, and microbiome influences [133,134,135,136]. The ability of zeolites to transition from promising multifunctional fillers to clinically validated dental materials will be determined by the combination of these phases.

## 4. Conclusions

Zeolites can be synthesized from industrial and agricultural byproducts, providing environmentally sustainable methods to produce fillers for use in dental materials. Zeolites with ion exchange capabilities through the incorporation of silver, zinc, and other ions offer bioactivity in terms of antimicrobial efficacy and anti-inflammatory and osteogenic potential. Moreover, regulated ion release from these zeolites enables the mitigation of secondary caries and infections near restoration margins. Calcium and strontium zeolites facilitate tooth remineralization and tissue regeneration. In addition, enhancing filler–polymer compatibility improves the mechanical strength, wear resistance, and durability of composites, adhesives, cements, and implant coatings. Zeolite-modified dental materials demonstrate potential in various applications, including resin cement, glass ionomers, root canal irrigants, and implant coatings. Nonetheless, further in vitro and in vivo investigations are required to validate the efficacy and safety of zeolite-enhanced dental materials.

## Figures and Tables

**Table 1:** Ion-exchange zeolites in dentistry: functions and applications.

Ion	Zeolite Frameworks Used	Dental Applications	Main Benefits	References
Silver (Ag^+^)	FAU (Zeolite X/Y), LTA, Na-A	Restorative composites, glass ionomer cements (GICs), mineral trioxide aggregate (MTA), resin cements, prosthetic acrylic polymers, and implant coatings	Sustained ion release prevents secondary caries; efficacious against *S. mutans*, *E. faecalis*, and *C. albicans*; broad-spectrum antimicrobial activity	[1,5,6,11]
Zinc (Zn^2+^)	LTA, Na-A, FAU	Dental adhesives, restorative composites, GICs, and implant coatings	inhibits dentin collagen degradation (MMP inhibition); exhibits antimicrobial, anti-inflammatory, and osteogenic properties	[2,3,4]
Calcium (Ca^2+^)	CAU, Na-A, LTA	Remineralizing additives in cements, adhesives, and composites	Enhances the remineralization of enamel/dentin, promotes hydroxyapatite regeneration, and aids in the prevention of caries	[16,40,90]
Strontium (Sr^2+^)	CAU, LTA	Restorative composites and regenerative materials	It serves a dual function by stimulating stem cells and remineralizing teeth, thereby improving the acid resistance of dental tissues.	[11,81,91]
Copper (Cu^2+^)	FAU, LTA	Restorative materials and antibacterial coatings	Strong antimicrobial effects, including those against resistant pathogens; can be combined with CuO nanoparticles to enhance their effectiveness	

**Table 2:** Comparison of zeolites with other emergent dental fillers.

Attribute	Zeolites	Bioactive Glass (BAG)	Hydroxyapatite Nanoparticles (nHA)	References
Primary function(s)	Drug delivery via ion exchange, remineralization (Ca, Sr), antimicrobial (Ag, Zn)	Hydroxycarbonate apatite layer formation; desensitization; Ca/Si/P release	“Biomimetic remineralization; osteointegration (coatings)”	[1,128,131,132,133,134,135]
Release profile	Risk of chronic exposure if uncontrolled; tunable/sustained	Frequently, the initial explosion is followed by a reduction.	Low unless functionalized or enhanced	[128,131,132,133,134]
Antimicrobial activity	Broad spectrum; high with Ag/Cu exchange	Mostly pH/ionic, moderate	Modest unless altered	[1,129,131,135]
Mechanical properties	Interface Key; load/dispersion-sensitive	High pressures may cause brittleness.	Aggregation may deteriorate; compatibility is necessary.	[129,131,135]
Clinical maturity	Predominantly preclinical/bench; limited clinical	Implemented in desensitizers; select restoratives	Early clinical application in coatings and pastes; a smaller number of dental products that have been approved	[131,135]

## Data Availability

The data set used and/or analyzed during the current study are available from the corresponding author upon reasonable request.
